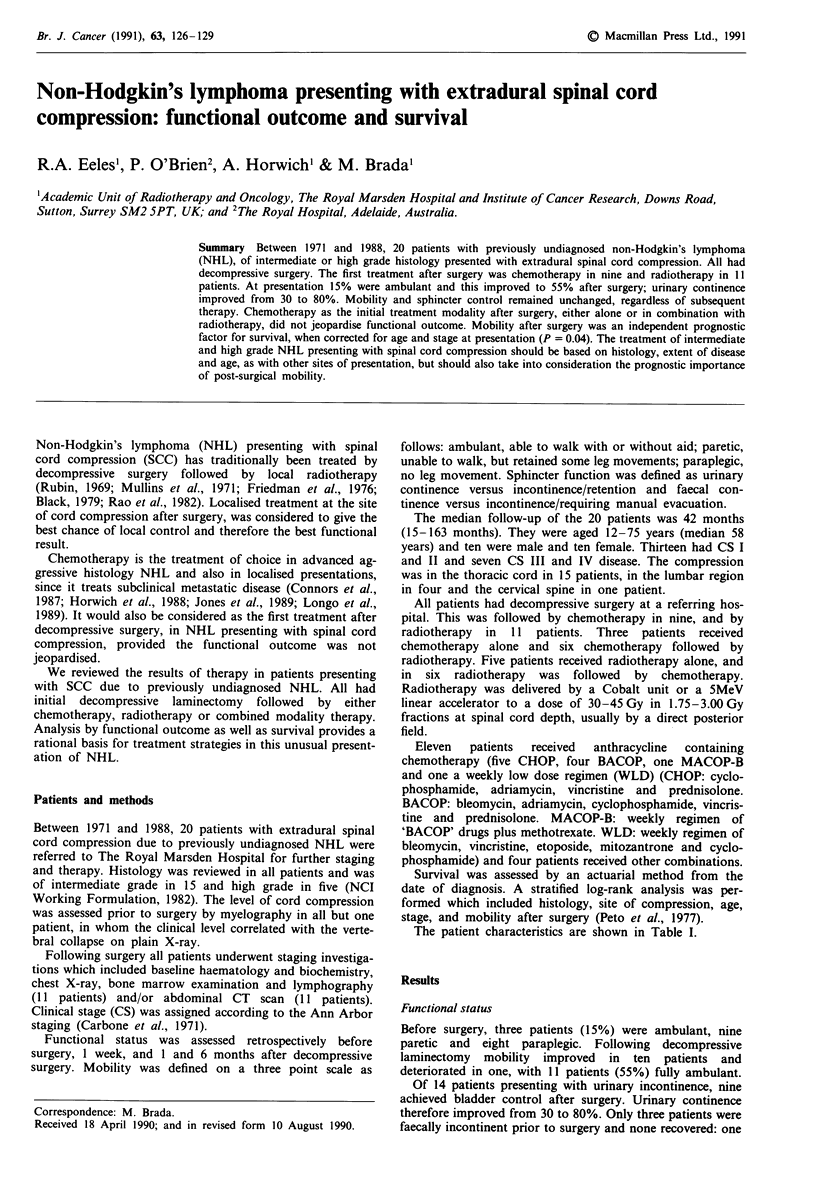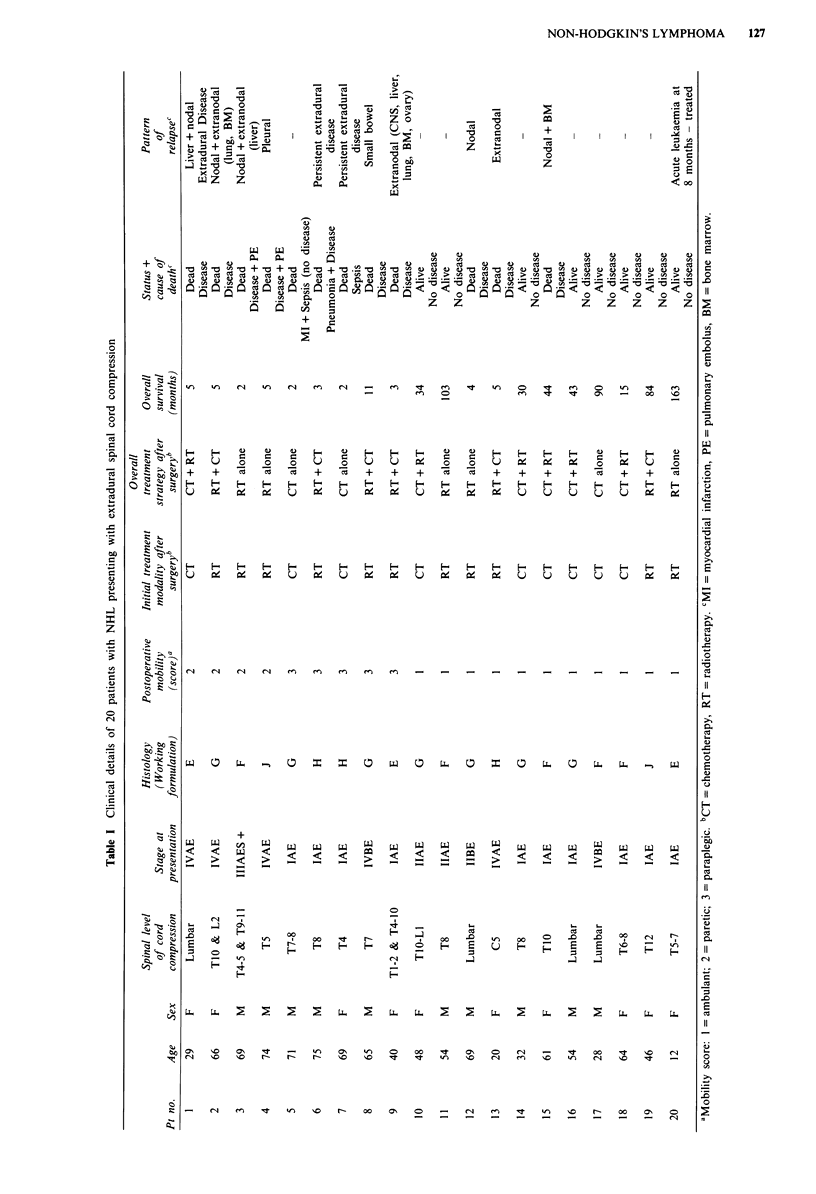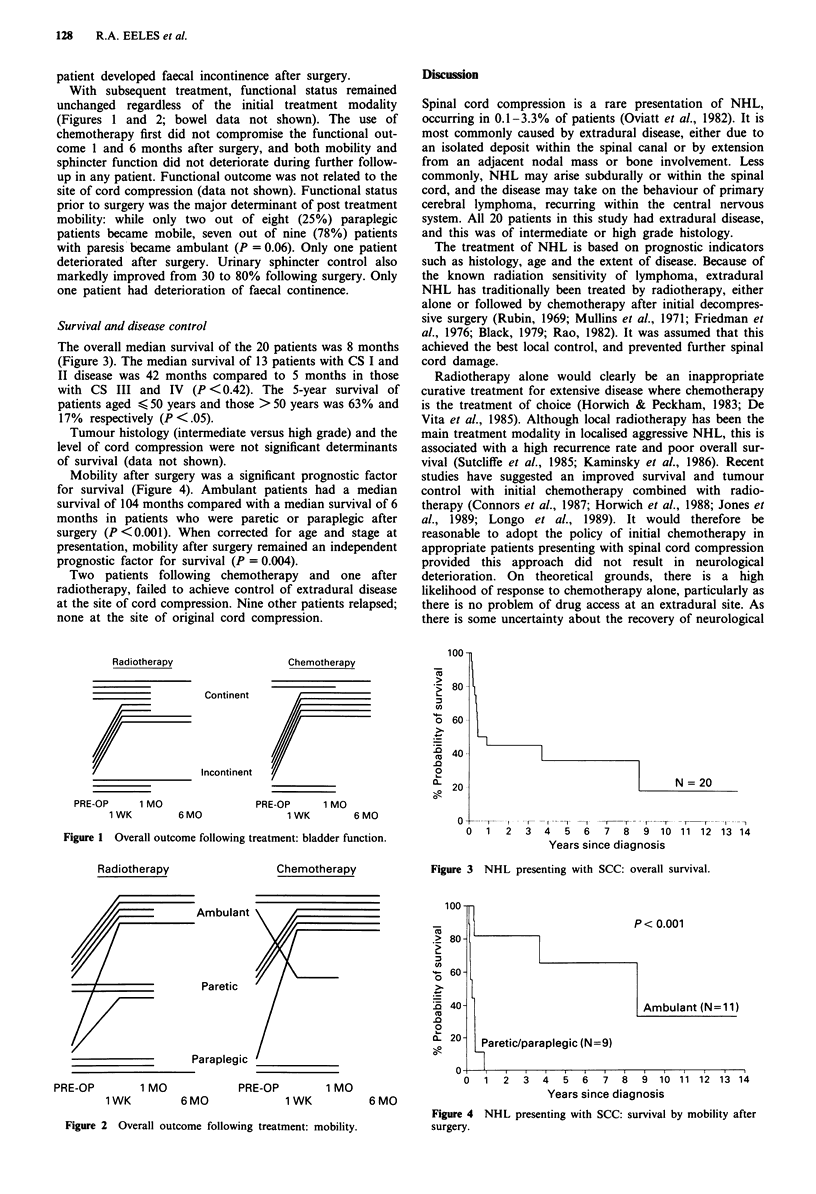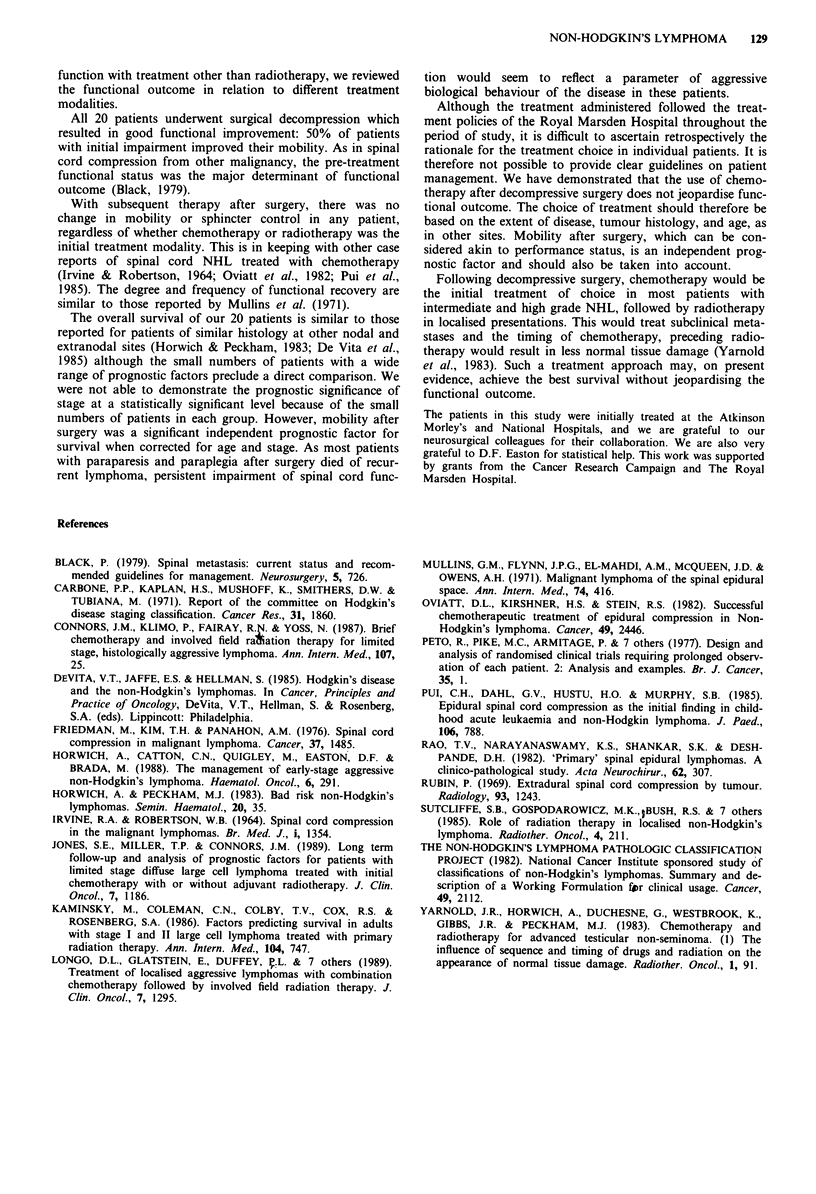# Non-Hodgkin's lymphoma presenting with extradural spinal cord compression: functional outcome and survival.

**DOI:** 10.1038/bjc.1991.25

**Published:** 1991-01

**Authors:** R. A. Eeles, P. O'Brien, A. Horwich, M. Brada

**Affiliations:** Academic Unit of Radiotherapy and Oncology, Royal Marsden Hospital, Adelaide, Australia.

## Abstract

Between 1971 and 1988, 20 patients with previously undiagnosed non-Hodgkin's lymphoma (NHL), of intermediate or high grade histology presented with extradural spinal cord compression. All had decompressive surgery. The first treatment after surgery was chemotherapy in nine and radiotherapy in 11 patients. At presentation 15% were ambulant and this improved to 55% after surgery; urinary continence improved from 30 to 80%. Mobility and sphincter control remained unchanged, regardless of subsequent therapy. Chemotherapy as the initial treatment modality after surgery, either alone or in combination with radiotherapy, did not jeopardise functional outcome. Mobility after surgery was an independent prognostic factor for survival, when corrected for age and stage at presentation (P = 0.04). The treatment of intermediate and high grade NHL presenting with spinal cord compression should be based on histology, extent of disease and age, as with other sites of presentation, but should also take into consideration the prognostic importance of post-surgical mobility.


Br. J. Cancer (1991), 63, 126-129 © Macmillan Press Ltd., 1991

### Non-Hodgkin's lymphoma presenting with extradural spinal cord compression: functional outcome and survival

R.A. Eeles[1], P. O'Brien[2], A. Horwich[1] & M. Brada[1]

[1]*Academic Unit of Radiotherapy and Oncology, The Royal Marsden Hospital and Institute of Cancer Research, Downs Road, Sutton, Surrey SM2 5PT, UK; and* [2]*The Royal Hospital, Adelaide, Australia.*

**Summary** Between 1971 and 1988, 20 patients with previously undiagnosed non-Hodgkin's lymphoma (NHL), of intermediate or high grade histology presented with extradural spinal cord compression. All had decompressive surgery. The first treatment after surgery was chemotherapy in nine and radiotherapy in 11 patients. At presentation 15% were ambulant and this improved to 55% after surgery; urinary continence improved from 30 to 80%. Mobility and sphincter control remained unchanged, regardless of subsequent therapy. Chemotherapy as the initial treatment modality after surgery, either alone or in combination with radiotherapy, did not jeopardise functional outcome. Mobility after surgery was an independent prognostic factor for survival, when corrected for age and stage at presentation ($P = 0.04$). The treatment of intermediate and high grade NHL presenting with spinal cord compression should be based on histology, extent of disease and age, as with other sites of presentation, but should also take into consideration the prognostic importance of post-surgical mobility.

Non-Hodgkin's lymphoma (NHL) presenting with spinal cord compression (SCC) has traditionally been treated by decompressive surgery followed by local radiotherapy (Rubin, 1969; Mullins *et al.*, 1971; Friedman *et al.*, 1976; Black, 1979; Rao *et al.*, 1982). Localised treatment at the site of cord compression after surgery, was considered to give the best chance of local control and therefore the best functional result.

Chemotherapy is the treatment of choice in advanced aggressive histology NHL and also in localised presentations, since it treats subclinical metastatic disease (Connors *et al.*, 1987; Horwich *et al.*, 1988; Jones *et al.*, 1989; Longo *et al.*, 1989). It would also be considered as the first treatment after decompressive surgery, in NHL presenting with spinal cord compression, provided the functional outcome was not jeopardised.

We reviewed the results of therapy in patients presenting with SCC due to previously undiagnosed NHL. All had initial decompressive laminectomy followed by either chemotherapy, radiotherapy or combined modality therapy. Analysis by functional outcome as well as survival provides a rational basis for treatment strategies in this unusual presentation of NHL.

### Patients and methods

Between 1971 and 1988, 20 patients with extradural spinal cord compression due to previously undiagnosed NHL were referred to The Royal Marsden Hospital for further staging and therapy. Histology was reviewed in all patients and was of intermediate grade in 15 and high grade in five (NCI Working Formulation, 1982). The level of cord compression was assessed prior to surgery by myelography in all but one patient, in whom the clinical level correlated with the vertebral collapse on plain X-ray.

Following surgery all patients underwent staging investigations which included baseline haematology and biochemistry, chest X-ray, bone marrow examination and lymphography (11 patients) and/or abdominal CT scan (11 patients). Clinical stage (CS) was assigned according to the Ann Arbor staging (Carbone *et al.*, 1971).

Functional status was assessed retrospectively before surgery, 1 week, and 1 and 6 months after decompressive surgery. Mobility was defined on a three point scale as follows: ambulant, able to walk with or without aid; paretic, unable to walk, but retained some leg movements; paraplegic, no leg movement. Sphincter function was defined as urinary continence versus incontinence/retention and faecal continence versus incontinence/requiring manual evacuation.

The median follow-up of the 20 patients was 42 months (15-163 months). They were aged 12-75 years (median 58 years) and ten were male and ten female. Thirteen had CS I and II and seven CS III and IV disease. The compression was in the thoracic cord in 15 patients, in the lumbar region in four and the cervical spine in one patient.

All patients had decompressive surgery at a referring hospital. This was followed by chemotherapy in nine, and by radiotherapy in 11 patients. Three patients received chemotherapy alone and six chemotherapy followed by radiotherapy. Five patients received radiotherapy alone, and in six radiotherapy was followed by chemotherapy. Radiotherapy was delivered by a Cobalt unit or a 5MeV linear accelerator to a dose of 30-45 Gy in 1.75-3.00 Gy fractions at spinal cord depth, usually by a direct posterior field.

Eleven patients received anthracycline containing chemotherapy (five CHOP, four BACOP, one MACOP-B and one a weekly low dose regimen (WLD) (CHOP: cyclophosphamide, adriamycin, vincristine and prednisolone. BACOP: bleomycin, adriamycin, cyclophosphamide, vincristine and prednisolone. MACOP-B: weekly regimen of 'BACOP' drugs plus methotrexate. WLD: weekly regimen of bleomycin, vincristine, etoposide, mitozantrone and cyclophosphamide) and four patients received other combinations.

Survival was assessed by an actuarial method from the date of diagnosis. A stratified log-rank analysis was performed which included histology, site of compression, age, stage, and mobility after surgery (Peto *et al.*, 1977).

The patient characteristics are shown in Table I.

### Results

### *Functional status*

Before surgery, three patients (15%) were ambulant, nine paretic and eight paraplegic. Following decompressive laminectomy mobility improved in ten patients and deteriorated in one, with 11 patients (55%) fully ambulant.

Of 14 patients presenting with urinary incontinence, nine achieved bladder control after surgery. Urinary continence therefore improved from 30 to 80%. Only three patients were faecally incontinent prior to surgery and none recovered: one

Correspondence: M. Brada.
Received 18 April 1990; and in revised form 10 August 1990.

**Table I** Clinical details of 20 patients with NHL presenting with extradural spinal cord compression

| *Pt no.* | *Age* | *Sex* | *Spinal level of cord compression* | *Stage at presentation* | *Histology (Working formulation)* | *Postoperative mobility (score)*[a] | *Initial treatment modality after surgery*[b] | *Overall treatment strategy after surgery*[b] | *Overall survival (months)* | *Status + cause of death*[c] | *Pattern of relapse*[c] |
|---|---|---|---|---|---|---|---|---|---|---|---|
| 1 | 29 | F | Lumbar | IVAE | E | 2 | CT | CT + RT | 5 | Dead Disease | Liver + nodal Extradural Disease |
| 2 | 66 | F | T10 & L2 | IVAE | G | 2 | RT | RT + CT | 5 | Dead Disease | Nodal + extranodal (lung, BM) |
| 3 | 69 | M | T4-5 & T9-11 | IIIAES + | F | 2 | RT | RT alone | 2 | Dead Disease + PE | Nodal + extranodal (liver) |
| 4 | 74 | M | T5 | IVAE | J | 2 | RT | RT alone | 5 | Dead Disease + PE | Pleural |
| 5 | 71 | M | T7-8 | IAE | G | 3 | CT | CT alone | 2 | Dead MI + Sepsis (no disease) | – |
| 6 | 75 | M | T8 | IAE | H | 3 | RT | RT + CT | 3 | Dead Pneumonia + Disease | Persistent extradural disease |
| 7 | 69 | F | T4 | IAE | H | 3 | CT | CT alone | 2 | Dead Sepsis | Persistent extradural disease |
| 8 | 65 | M | T7 | IVBE | G | 3 | RT | RT + CT | 11 | Dead Disease | Small bowel |
| 9 | 40 | F | T1-2 & T4-10 | IAE | E | 3 | RT | RT + CT | 3 | Dead Disease | Extranodal (CNS, liver, lung, BM, ovary) |
| 10 | 48 | F | T10-L1 | IIAE | G | 1 | CT | CT + RT | 34 | Alive No disease | – |
| 11 | 54 | M | T8 | IIAE | F | 1 | RT | RT alone | 103 | Alive No disease | – |
| 12 | 69 | M | Lumbar | IIBE | G | 1 | RT | RT alone | 4 | Dead Disease | Nodal |
| 13 | 20 | F | C5 | IVAE | H | 1 | RT | RT + CT | 5 | Dead Disease | Extranodal |
| 14 | 32 | M | T8 | IAE | G | 1 | CT | CT + RT | 30 | Alive No disease | – |
| 15 | 61 | F | T10 | IAE | F | 1 | CT | CT + RT | 44 | Dead Disease | Nodal + BM |
| 16 | 54 | M | Lumbar | IAE | G | 1 | CT | CT + RT | 43 | Alive No disease | – |
| 17 | 28 | M | Lumbar | IVBE | F | 1 | CT | CT alone | 90 | Alive No disease | – |
| 18 | 64 | F | T6-8 | IAE | F | 1 | CT | CT + RT | 15 | Alive No disease | – |
| 19 | 46 | F | T12 | IAE | J | 1 | RT | RT + CT | 84 | Alive No disease | – |
| 20 | 12 | F | T5-7 | IAE | E | 1 | RT | RT alone | 163 | Alive No disease | Acute leukaemia at 8 months – treated |

[a]Mobility score: 1 = ambulant; 2 = paretic; 3 = paraplegic. [b]CT = chemotherapy, RT = radiotherapy. [c]MI = myocardial infarction, PE = pulmonary embolus, BM = bone marrow.

patient developed faecal incontinence after surgery.

With subsequent treatment, functional status remained unchanged regardless of the initial treatment modality (Figures 1 and 2; bowel data not shown). The use of chemotherapy first did not compromise the functional outcome 1 and 6 months after surgery, and both mobility and sphincter function did not deteriorate during further follow-up in any patient. Functional outcome was not related to the site of cord compression (data not shown). Functional status prior to surgery was the major determinant of post treatment mobility: while only two out of eight (25%) paraplegic patients became mobile, seven out of nine (78%) patients with paresis became ambulant ($P = 0.06$). Only one patient deteriorated after surgery. Urinary sphincter control also markedly improved from 30 to 80% following surgery. Only one patient had deterioration of faecal continence.

### *Survival and disease control*

The overall median survival of the 20 patients was 8 months (Figure 3). The median survival of 13 patients with CS I and II disease was 42 months compared to 5 months in those with CS III and IV ($P < 0.42$). The 5-year survival of patients aged $\leqslant 50$ years and those $> 50$ years was 63% and 17% respectively ($P < .05$).

Tumour histology (intermediate versus high grade) and the level of cord compression were not significant determinants of survival (data not shown).

Mobility after surgery was a significant prognostic factor for survival (Figure 4). Ambulant patients had a median survival of 104 months compared with a median survival of 6 months in patients who were paretic or paraplegic after surgery ($P < 0.001$). When corrected for age and stage at presentation, mobility after surgery remained an independent prognostic factor for survival ($P = 0.004$).

Two patients following chemotherapy and one after radiotherapy, failed to achieve control of extradural disease at the site of cord compression. Nine other patients relapsed; none at the site of original cord compression.

### Discussion

Spinal cord compression is a rare presentation of NHL, occurring in 0.1–3.3% of patients (Oviatt *et al.*, 1982). It is most commonly caused by extradural disease, either due to an isolated deposit within the spinal canal or by extension from an adjacent nodal mass or bone involvement. Less commonly, NHL may arise subdurally or within the spinal cord, and the disease may take on the behaviour of primary cerebral lymphoma, recurring within the central nervous system. All 20 patients in this study had extradural disease, and this was of intermediate or high grade histology.

The treatment of NHL is based on prognostic indicators such as histology, age and the extent of disease. Because of the known radiation sensitivity of lymphoma, extradural NHL has traditionally been treated by radiotherapy, either alone or followed by chemotherapy after initial decompressive surgery (Rubin, 1969; Mullins *et al.*, 1971; Friedman *et al.*, 1976; Black, 1979; Rao, 1982). It was assumed that this achieved the best local control, and prevented further spinal cord damage.

Radiotherapy alone would clearly be an inappropriate curative treatment for extensive disease where chemotherapy is the treatment of choice (Horwich & Peckham, 1983; De Vita *et al.*, 1985). Although local radiotherapy has been the main treatment modality in localised aggressive NHL, this is associated with a high recurrence rate and poor overall survival (Sutcliffe *et al.*, 1985; Kaminsky *et al.*, 1986). Recent studies have suggested an improved survival and tumour control with initial chemotherapy combined with radiotherapy (Connors *et al.*, 1987; Horwich *et al.*, 1988; Jones *et al.*, 1989; Longo *et al.*, 1989). It would therefore be reasonable to adopt the policy of initial chemotherapy in appropriate patients presenting with spinal cord compression provided this approach did not result in neurological deterioration. On theoretical grounds, there is a high likelihood of response to chemotherapy alone, particularly as there is no problem of drug access at an extradural site. As there is some uncertainty about the recovery of neurological

**Figure 1** Overall outcome following treatment: bladder function.

**Figure 2** Overall outcome following treatment: mobility.

**Figure 3** NHL presenting with SCC: overall survival.

**Figure 4** NHL presenting with SCC: survival by mobility after surgery.

function with treatment other than radiotherapy, we reviewed the functional outcome in relation to different treatment modalities.

All 20 patients underwent surgical decompression which resulted in good functional improvement: 50% of patients with initial impairment improved their mobility. As in spinal cord compression from other malignancy, the pre-treatment functional status was the major determinant of functional outcome (Black, 1979).

With subsequent therapy after surgery, there was no change in mobility or sphincter control in any patient, regardless of whether chemotherapy or radiotherapy was the initial treatment modality. This is in keeping with other case reports of spinal cord NHL treated with chemotherapy (Irvine & Robertson, 1964; Oviatt *et al.*, 1982; Pui *et al.*, 1985). The degree and frequency of functional recovery are similar to those reported by Mullins *et al.* (1971).

The overall survival of our 20 patients is similar to those reported for patients of similar histology at other nodal and extranodal sites (Horwich & Peckham, 1983; De Vita *et al.*, 1985) although the small numbers of patients with a wide range of prognostic factors preclude a direct comparison. We were not able to demonstrate the prognostic significance of stage at a statistically significant level because of the small numbers of patients in each group. However, mobility after surgery was a significant independent prognostic factor for survival when corrected for age and stage. As most patients with paraparesis and paraplegia after surgery died of recurrent lymphoma, persistent impairment of spinal cord function would seem to reflect a parameter of aggressive biological behaviour of the disease in these patients.

Although the treatment administered followed the treatment policies of the Royal Marsden Hospital throughout the period of study, it is difficult to ascertain retrospectively the rationale for the treatment choice in individual patients. It is therefore not possible to provide clear guidelines on patient management. We have demonstrated that the use of chemotherapy after decompressive surgery does not jeopardise functional outcome. The choice of treatment should therefore be based on the extent of disease, tumour histology, and age, as in other sites. Mobility after surgery, which can be considered akin to performance status, is an independent prognostic factor and should also be taken into account.

Following decompressive surgery, chemotherapy would be the initial treatment of choice in most patients with intermediate and high grade NHL, followed by radiotherapy in localised presentations. This would treat subclinical metastases and the timing of chemotherapy, preceding radiotherapy would result in less normal tissue damage (Yarnold *et al.*, 1983). Such a treatment approach may, on present evidence, achieve the best survival without jeopardising the functional outcome.

The patients in this study were initially treated at the Atkinson Morley's and National Hospitals, and we are grateful to our neurosurgical colleagues for their collaboration. We are also very grateful to D.F. Easton for statistical help. This work was supported by grants from the Cancer Research Campaign and The Royal Marsden Hospital.

### References

BLACK, P. (1979). Spinal metastasis: current status and recommended guidelines for management. *Neurosurgery*, **5**, 726.

CARBONE, P.P., KAPLAN, H.S., MUSHOFF, K., SMITHERS, D.W. & TUBIANA, M. (1971). Report of the committee on Hodgkin's disease staging classification. *Cancer Res.*, **31**, 1860.

CONNORS, J.M., KLIMO, P., FAIRAY, R.N. & YOSS, N. (1987). Brief chemotherapy and involved field radiation therapy for limited stage, histologically aggressive lymphoma. *Ann. Intern. Med.*, **107**, 25.

DEVITA, V.T., JAFFE, E.S. & HELLMAN, S. (1985). Hodgkin's disease and the non-Hodgkin's lymphomas. In *Cancer, Principles and Practice of Oncology*, DeVita, V.T., Hellman, S. & Rosenberg, S.A. (eds). Lippincott: Philadelphia.

FRIEDMAN, M., KIM, T.H. & PANAHON, A.M. (1976). Spinal cord compression in malignant lymphoma. *Cancer*, **37**, 1485.

HORWICH, A., CATTON, C.N., QUIGLEY, M., EASTON, D.F. & BRADA, M. (1988). The management of early-stage aggressive non-Hodgkin's lymphoma. *Haematol. Oncol.*, **6**, 291.

HORWICH, A. & PECKHAM, M.J. (1983). Bad risk non-Hodgkin's lymphomas. *Semin. Haematol.*, **20**, 35.

IRVINE, R.A. & ROBERTSON, W.B. (1964). Spinal cord compression in the malignant lymphomas. *Br. Med. J.*, **i**, 1354.

JONES, S.E., MILLER, T.P. & CONNORS, J.M. (1989). Long term follow-up and analysis of prognostic factors for patients with limited stage diffuse large cell lymphoma treated with initial chemotherapy with or without adjuvant radiotherapy. *J. Clin. Oncol.*, **7**, 1186.

KAMINSKY, M., COLEMAN, C.N., COLBY, T.V., COX, R.S. & ROSENBERG, S.A. (1986). Factors predicting survival in adults with stage I and II large cell lymphoma treated with primary radiation therapy. *Ann. Intern. Med.*, **104**, 747.

LONGO, D.L., GLATSTEIN, E., DUFFEY, P.L. & 7 others (1989). Treatment of localised aggressive lymphomas with combination chemotherapy followed by involved field radiation therapy. *J. Clin. Oncol.*, **7**, 1295.

MULLINS, G.M., FLYNN, J.P.G., EL-MAHDI, A.M., MCQUEEN, J.D. & OWENS, A.H. (1971). Malignant lymphoma of the spinal epidural space. *Ann. Intern. Med.*, **74**, 416.

OVIATT, D.L., KIRSHNER, H.S. & STEIN, R.S. (1982). Successful chemotherapeutic treatment of epidural compression in Non-Hodgkin's lymphoma. *Cancer*, **49**, 2446.

PETO, R., PIKE, M.C., ARMITAGE, P. & 7 others (1977). Design and analysis of randomised clinical trials requiring prolonged observation of each patient. 2: Analysis and examples. *Br. J. Cancer*, **35**, 1.

PUI, C.H., DAHL, G.V., HUSTU, H.O. & MURPHY, S.B. (1985). Epidural spinal cord compression as the initial finding in childhood acute leukaemia and non-Hodgkin lymphoma. *J. Paed.*, **106**, 788.

RAO, T.V., NARAYANASWAMY, K.S., SHANKAR, S.K. & DESHPANDE, D.H. (1982). 'Primary' spinal epidural lymphomas. A clinico-pathological study. *Acta Neurochirur.*, **62**, 307.

RUBIN, P. (1969). Extradural spinal cord compression by tumour. *Radiology*, **93**, 1243.

SUTCLIFFE, S.B., GOSPODAROWICZ, M.K., BUSH, R.S. & 7 others (1985). Role of radiation therapy in localised non-Hodgkin's lymphoma. *Radiother. Oncol.*, **4**, 211.

THE NON-HODGKIN'S LYMPHOMA PATHOLOGIC CLASSIFICATION PROJECT (1982). National Cancer Institute sponsored study of classifications of non-Hodgkin's lymphomas. Summary and description of a Working Formulation for clinical usage. *Cancer*, **49**, 2112.

YARNOLD, J.R., HORWICH, A., DUCHESNE, G., WESTBROOK, K., GIBBS, J.R. & PECKHAM, M.J. (1983). Chemotherapy and radiotherapy for advanced testicular non-seminoma. (1) The influence of sequence and timing of drugs and radiation on the appearance of normal tissue damage. *Radiother. Oncol.*, **1**, 91.